# Post-spinal Aseptic Meningitis Following Cesarean Delivery Presenting as an Acute Febrile Meningeal Syndrome: A Diagnostic Challenge

**DOI:** 10.7759/cureus.117914

**Published:** 2026-10-14

**Authors:** Yasser Ouatab, Youssra Kramchi, Hassan Kraitiss, Marouane Jidal, Youssef Halhoul, Nabil Elachhab, Rajae Tachinante

**Affiliations:** 1 Anesthesia and Intensive Care, Mohammed V Military Training Hospital, Rabat, MAR; 2 Maternal Critical Care and Anesthesiology, Centre Hospitalo-Universitaire (CHU) Ibn Sina, Rabat, MAR; 3 Anesthesia and Critical Care, Maternity Hospital, Ibn Sina University Hospital, Rabat, MAR

**Keywords:** aseptic meningitis, cerebrospinal fluid, cesarean delivery, diplopia, meningeal syndrome, neuraxial anesthesia, postpartum, spinal anesthesia

## Abstract

Post-spinal aseptic meningitis is a rare complication of neuraxial anesthesia that may be clinically indistinguishable from bacterial meningitis at presentation. We report the case of an 18-year-old primigravida at approximately 38-39 weeks of gestation who underwent emergency cesarean delivery for acute fetal distress under spinal anesthesia with preservative-free hyperbaric bupivacaine and fentanyl. Within hours of the procedure, she developed somnolence followed by fever, confusion, neck stiffness, and photophobia. Cerebrospinal fluid (CSF) analysis revealed marked neutrophilic pleocytosis and substantial protein elevation with preserved glucose, while Gram staining and cultures remained negative. Empirical antimicrobial therapy was initiated because bacterial meningitis could not initially be excluded. During the subsequent course, the patient developed transient binocular diplopia with limitation of upward gaze. Brain and orbital magnetic resonance imaging showed no explanatory abnormalities. An extensive microbiological investigation, including bacterial, mycobacterial, fungal, and viral testing, remained negative. Although herpes simplex virus type 1 was detected from a subsequently appearing perioral lesion, CSF herpes simplex virus polymerase chain reaction was negative. Repeat lumbar puncture on day 10 demonstrated near-complete normalization of CSF findings, and the patient achieved complete neurological recovery, including resolution of diplopia. The close temporal relationship with spinal anesthesia, repeatedly negative microbiological investigations, normal neuroimaging, rapid CSF normalization, and favorable neurological outcome supported the diagnosis of post-spinal aseptic meningitis. This case emphasizes the importance of initially treating suspected post-neuraxial meningitis as a bacterial infection while considering aseptic meningitis when microbiological studies remain negative and the subsequent clinical and biological course supports a noninfectious inflammatory process.

## Introduction

Spinal anesthesia is widely used for cesarean delivery because it provides rapid and reliable surgical anesthesia while avoiding many of the risks associated with general anesthesia. Although serious neurological complications are uncommon, meningitis following neuraxial anesthesia represents a rare but potentially life-threatening event requiring prompt recognition and treatment [1,2].

Meningitis occurring after spinal anesthesia may result from bacterial contamination or, less commonly, from a sterile inflammatory reaction involving intrathecal drugs or inadvertent exposure to chemical irritants [1-6]. Distinguishing bacterial meningitis from aseptic or chemical meningitis at initial presentation can be particularly difficult because both conditions may manifest with fever, severe headache, neck stiffness, altered mental status, and marked cerebrospinal fluid (CSF) pleocytosis with neutrophilic predominance [1-6]. Consequently, suspected bacterial meningitis requires prompt empirical antimicrobial therapy while microbiological investigations are pending [7].

## Case presentation

An 18-year-old primigravida at approximately 38-39 weeks of gestation, with no significant medical or neurological history, was admitted in labor. Her pregnancy had been uneventful. Emergency cesarean delivery was indicated for acute fetal distress. The membranes had ruptured approximately two hours before surgery, with no documented maternal fever or clinical evidence of infection before the procedure.

At 10:00 AM, single-shot spinal anesthesia was performed at the L4-L5 interspace using a 25-gauge Vygon spinal needle after skin antisepsis with povidone-iodine. The procedure required two attempts. Preservative-free hyperbaric bupivacaine 12.5 mg combined with preservative-free fentanyl 25 µg was administered intrathecally. Adequate surgical anesthesia was obtained, and cesarean delivery with birth occurred at approximately 10:05 AM without an immediate anesthetic complication.

At approximately 2:00 PM, four hours after spinal anesthesia, the patient developed somnolence. By approximately 4:00 PM, her neurological condition had worsened, with confusion associated with fever and clinical features of an acute meningeal syndrome. Neck stiffness and photophobia were subsequently documented. No focal motor deficit was initially identified. The chronological sequence of the clinical course is summarized in Figure 1.

**Figure 1:**
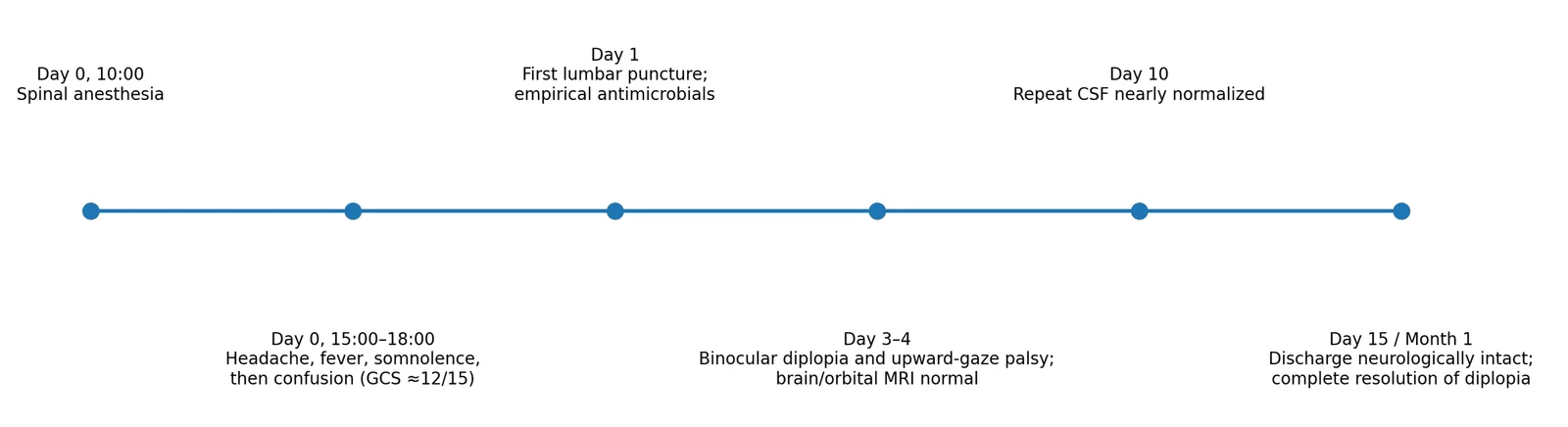
Timeline of the clinical course following spinal anesthesia The timeline summarizes the onset of neurological symptoms following spinal anesthesia, the diagnostic investigations, the development of binocular diplopia, repeat CSF analysis, and subsequent neurological recovery. CSF: cerebrospinal fluid; GCS: Glasgow Coma Scale; MRI: magnetic resonance imaging

Initial systemic laboratory investigations demonstrated marked neutrophilic leukocytosis, with a white blood cell count of 30.1 × 10⁹/L (normal range: 4.0-10.0 × 10⁹/L) and an absolute neutrophil count of 28.7 × 10⁹/L (normal range: 1.5-7.5 × 10⁹/L), representing 95.6%. C-reactive protein was 25.2 mg/L (normal range: <5 mg/L), platelet count was 233 × 10⁹/L (normal range: 150-400 × 10⁹/L), serum creatinine was 5.8 mg/L (normal range: 6-11 mg/L), aspartate aminotransferase was 38 U/L (normal range: 10-40 U/L), and alanine aminotransferase was 7 U/L (normal range: 7-40 U/L). Procalcitonin was not available at the initial presentation.

According to the available medical record, urgent non-contrast brain computed tomography (CT) and subsequent vascular imaging showed no acute intracranial or vascular abnormality. The original imaging files were no longer available for review or inclusion in this report. Cerebral venous thrombosis was considered in the postpartum differential diagnosis, and anticoagulation was initiated. Consequently, lumbar puncture was postponed for approximately 12 hours and performed after an appropriate interval from anticoagulation.

The first CSF examination demonstrated a markedly inflammatory profile. The available laboratory records reported marked pleocytosis with neutrophilic predominance, with a leukocyte count reaching approximately 2,000 cells/mm³ (normal range: 0-5 cells/mm³), protein concentration of approximately 3.05 g/L (normal range: 0.15-0.45 g/L), and preserved CSF glucose. Gram staining was negative, and bacterial cultures remained sterile. Opening pressure was not recorded.

The initial lumbar puncture was performed before the administration of empirical antimicrobial therapy and corticosteroids. Following CSF collection, intravenous antibiotic therapy was initiated because bacterial meningitis could not be excluded. The treatment regimen included ceftriaxone for 10 days, levofloxacin 500 mg/day for 10 days, and gentamicin 160 mg/day for five days. Corticosteroid therapy was initiated concomitantly with antibiotics and continued for four days. The exact ceftriaxone dosage could not be reliably verified from the available medical records.

During the subsequent clinical course, intravenous acyclovir was administered for 21 days because herpetic meningoencephalitis was considered in the differential diagnosis. Although herpes simplex virus type 1 (HSV-1) was detected in a perioral lesion, CSF HSV polymerase chain reaction (PCR) was negative. Empirical antituberculous therapy with ERIP-K4 (four tablets daily) was temporarily initiated while tuberculosis was being investigated.

An extensive microbiological evaluation was performed. Blood and CSF bacterial cultures remained negative. CSF investigations for *Mycobacterium tuberculosis*, fungal infection, and viral causes were unrevealing. In particular, CSF HSV PCR was negative.

On postoperative day 4, the patient developed binocular diplopia associated with limitation of upward gaze. Ophthalmological assessment raised concern for possible herpetic meningoencephalitis and prompted further neuroimaging. On postoperative day 5, brain and orbital magnetic resonance imaging (MRI) were reported as normal, without an identified intracranial or orbital abnormality explaining the ocular motor disturbance. A detailed neuro-ophthalmological examination was not available in the medical record.

During the subsequent course, perioral vesicular lesions appeared, and PCR testing of the lesion detected HSV-1. Because viral meningoencephalitis remained a consideration at that stage, intravenous acyclovir was initiated. However, the absence of HSV DNA in the CSF and the normal brain MRI did not support central nervous system HSV infection.

Serial systemic laboratory testing showed progressive improvement. C-reactive protein decreased to 10.8 mg/L and subsequently to 4.1 mg/L (normal range: <5 mg/L). Procalcitonin, when subsequently measured, decreased from 0.46 ng/mL to 0.05 ng/mL (normal range: <0.5 ng/mL). The white blood cell count decreased to 12.8 × 10⁹/L (normal range: 4.0-10.0 × 10⁹/L), with an absolute neutrophil count of 9.1 × 10⁹/L (normal range: 1.5-7.5 × 10⁹/L). Renal and hepatic indices remained reassuring during follow-up. The evolution of the main systemic laboratory parameters during hospitalization is summarized in Table 1.

**Table 1:** Evolution of systemic laboratory findings during hospitalization Serial systemic laboratory findings showing the initial inflammatory response and subsequent improvement during hospitalization. Reference ranges are provided in parentheses. "Not available" indicates that the corresponding value was not available in the medical record.

Parameter (reference range)	Initial value	Follow-up value(s)
White blood cell count (4.0-10.0 × 10⁹/L)	30.1 × 10⁹/L	12.8 × 10⁹/L
Absolute neutrophil count (1.5-7.5 × 10⁹/L)	28.7 × 10⁹/L	9.1 × 10⁹/L
C-reactive protein (<5 mg/L)	25.2 mg/L	10.8 mg/L → 4.1 mg/L
Procalcitonin (<0.5 ng/mL)	Not available	0.46 ng/mL → 0.05 ng/mL
Platelet count (150-400 × 10⁹/L)	233 × 10⁹/L	306 × 10⁹/L
Serum creatinine (6-11 mg/L)	5.8 mg/L	4.4-4.9 mg/L
Aspartate aminotransferase (10-40 U/L)	38 U/L	13-18 U/L
Alanine aminotransferase (7-40 U/L)	7 U/L	7-12 U/L

A repeat lumbar puncture performed on day 10 demonstrated near-complete resolution of the CSF inflammatory abnormalities, with 3 leukocytes/µL (normal range: 0-5 cells/µL), protein concentration of 0.17 g/L (normal range: 0.15-0.45 g/L), preserved glucose, negative Gram staining, and sterile culture. This rapid biological improvement, together with repeatedly negative microbiological investigations and the absence of an acute explanatory abnormality on the available neuroimaging reports, supported a nonbacterial inflammatory meningeal process. Serial CSF findings are summarized in Table 2.

**Table 2:** Evolution of CSF findings between the initial and repeat lumbar punctures The initial CSF showed marked neutrophilic inflammation with elevated protein and preserved glucose, whereas repeat CSF analysis on day 10 demonstrated near-complete normalization with persistently negative microbiological studies. CSF: cerebrospinal fluid

Parameter (reference range)	Initial lumbar puncture	Repeat lumbar puncture (day 10)
Leukocyte count (0-5 cells/µL)	~2,000 cells/µL	3 cells/µL
Leukocyte predominance	Neutrophilic	Not available
Protein (0.15-0.45 g/L)	3.05 g/L	0.17 g/L
Glucose	Preserved	Preserved
Red blood cell count (0-5 cells/µL)	Not available	90 cells/µL
Gram stain	Negative	Negative
Bacterial culture	Negative	Negative

The patient's clinical condition progressively improved, with resolution of fever, meningeal signs, and altered consciousness. She was discharged neurologically stable on day 15. The binocular diplopia subsequently resolved completely, and at the one-month follow-up, no residual neurological deficit was documented. Considering the close temporal relationship with spinal anesthesia, the sterile microbiological workup, the rapid normalization of CSF findings, the absence of an acute explanatory abnormality in the available neuroimaging reports, and the complete neurological recovery, the final diagnosis was post-spinal aseptic meningitis.

## Discussion

Post-spinal meningitis is a rare but potentially serious complication of neuraxial anesthesia. The initial distinction between bacterial meningitis and aseptic or chemical meningitis may be particularly difficult because the clinical manifestations and CSF abnormalities can overlap considerably. Fever, headache, neck stiffness, altered consciousness, neutrophilic pleocytosis, and elevated CSF protein may occur in both conditions [1-6]. Therefore, bacterial meningitis should remain the initial working diagnosis when a patient develops an acute febrile meningeal syndrome after spinal anesthesia, and empirical antimicrobial treatment should not be delayed while microbiological investigations are pending [7].

In the present case, several features initially raised concern for bacterial meningitis. The patient developed fever, altered consciousness, neck stiffness, and photophobia within hours of spinal anesthesia, while the first CSF analysis showed marked neutrophilic pleocytosis and substantial protein elevation. However, the subsequent course progressively argued against a bacterial etiology. Gram staining and CSF cultures remained negative, blood cultures did not identify a causative organism, extensive microbiological investigations were unrevealing, the available neuroimaging reports did not identify an acute explanatory abnormality, and repeat CSF analysis showed near-complete normalization by day 10. The patient ultimately achieved complete neurological recovery. Taken together, these findings supported the diagnosis of post-spinal aseptic meningitis, which remains fundamentally a diagnosis of exclusion [1-5].

Aseptic meningitis associated with spinal anesthesia has previously been described after intrathecal local anesthetic administration, particularly bupivacaine [1-3]. Proposed mechanisms include direct chemical irritation of the meninges by an intrathecal agent, contamination by trace chemical substances, and an inflammatory or hypersensitivity response to drugs introduced into the subarachnoid space [1,4,5]. Drug-induced aseptic meningitis may produce a striking neutrophilic CSF response and marked protein elevation, thereby closely mimicking bacterial meningitis [2-5]. In our patient, both hyperbaric bupivacaine and fentanyl were preservative-free, and no direct evidence of chemical contamination was identified. Consequently, although the temporal relationship with spinal anesthesia suggests a procedure-related sterile inflammatory reaction, causality cannot be attributed to a specific drug or agent.

The short interval between spinal anesthesia and symptom onset is noteworthy. Symptoms in our patient began approximately four hours after the neuraxial procedure, with progression to a febrile meningeal syndrome within the following hours. Previous reports of aseptic or chemical meningitis following spinal anesthesia have similarly described rapid onset. Tateno et al. reported severe headache and high fever shortly after cesarean delivery under intrathecal bupivacaine, with CSF showing 3,040 cells/mm³, predominantly polymorphonuclear cells, and markedly elevated protein [3]. Doghmi et al. described a 31-year-old woman who developed severe headache, neck stiffness, agitation, and subsequent loss of consciousness approximately 10 hours after spinal anesthesia with bupivacaine for cesarean delivery [1]. Oliveira et al. subsequently reported two cases of drug-induced aseptic meningitis after spinal anesthesia in which symptoms developed shortly after exposure, microbiological studies were negative, and both patients recovered without neurological sequelae [2]. These observations demonstrate that rapid symptom onset combined with an inflammatory but sterile CSF profile should raise suspicion for aseptic meningitis, although timing alone cannot safely exclude bacterial infection.

The principal differential diagnoses considered during the diagnostic workup are summarized in Table 3.

**Table 3:** Differential diagnosis of acute febrile meningeal syndrome following spinal anesthesia Principal differential diagnoses considered during the diagnostic workup and the clinical, microbiological, radiological, and CSF findings supporting or arguing against each diagnosis. CSF: cerebrospinal fluid; HSV: herpes simplex virus; PCR: polymerase chain reaction; MRI: magnetic resonance imaging

Differential diagnosis	Findings supporting consideration	Findings arguing against the diagnosis
Bacterial meningitis	Acute febrile meningeal syndrome; altered consciousness; marked neutrophilic CSF pleocytosis; elevated CSF protein	Repeatedly negative Gram stain and cultures; preserved CSF glucose; rapid clinical and CSF improvement
Post-spinal aseptic/chemical meningitis	Close temporal relationship with spinal anesthesia; rapid symptom onset; neutrophilic CSF pleocytosis; sterile microbiological investigations; rapid CSF normalization	No definitive biomarker; no specific causative drug or chemical agent demonstrated
Cerebral venous thrombosis	Postpartum setting; acute neurological deterioration	Vascular imaging did not identify a vascular explanation
HSV meningoencephalitis	Altered consciousness; subsequent perioral HSV-1-positive lesions	CSF HSV PCR negative; brain MRI did not demonstrate an explanatory encephalitic lesion
Tuberculous meningitis	Considered during the diagnostic workup	Tuberculosis investigations negative; rapid clinical and CSF improvement

Acute bacterial meningitis remained the most important diagnosis to exclude because failure to initiate timely treatment can result in severe neurological morbidity or death [7]. Moreover, post-dural puncture bacterial meningitis is a recognized complication of neuraxial procedures, with contamination from oropharyngeal or cutaneous flora among the reported mechanisms [6]. The absence of an isolated microorganism alone is insufficient to exclude bacterial meningitis, particularly when antimicrobial treatment has already been administered. In the present case, however, the combination of repeatedly negative microbiological investigations, preserved CSF glucose, rapid clinical improvement, substantial normalization of CSF parameters, and complete neurological recovery favored an aseptic inflammatory process.

Cerebral venous thrombosis was another important consideration because of the postpartum setting and the acute neurological deterioration. Anticoagulation was therefore initially administered, resulting in an approximately 12-hour delay before lumbar puncture could be safely performed. Subsequent vascular imaging did not identify a vascular explanation for the neurological syndrome. Other postpartum neurological disorders, including posterior reversible encephalopathy syndrome and ischemic or hemorrhagic cerebrovascular disease, were also considered but were not supported by the neuroimaging findings or subsequent clinical course.

HSV infection represented an additional diagnostic challenge. The subsequent appearance of perioral vesicular lesions with HSV-1 detected by PCR prompted the consideration of herpetic meningoencephalitis and justified empirical acyclovir while the diagnostic evaluation was ongoing. However, CSF HSV PCR was negative, brain MRI did not demonstrate an explanatory encephalitic lesion, and the subsequent clinical and biological evolution was not supportive of central nervous system HSV infection. The positive mucocutaneous HSV-1 result was therefore interpreted as evidence of peripheral viral reactivation rather than proof of HSV meningoencephalitis.

Another distinctive feature of this case was the development of binocular diplopia with limitation of upward gaze on postoperative day 4. Ocular motor disturbances may occur following dural puncture, classically in association with intracranial hypotension and most commonly involving the abducens nerve. In this patient, however, brain and orbital MRI were reported as normal, and no structural explanation for the vertical gaze disturbance was identified. The ocular symptoms subsequently resolved completely. Because a detailed neuro-ophthalmological examination was not available, the precise anatomical mechanism cannot be established. Nevertheless, the transient ocular motor abnormality broadens the neurological spectrum observed during this episode and represents an unusual feature of the present case.

The clinical characteristics of selected previously reported cases of aseptic or chemical meningitis following spinal anesthesia are compared with the present case in Table 4.

**Table 4:** Comparison of selected published cases of aseptic or chemical meningitis following spinal anesthesia Comparison of selected published cases of aseptic or chemical meningitis following spinal anesthesia with the present case, including clinical setting, intrathecal agents, symptom onset, CSF findings, neuroimaging, treatment, and outcome. CSF: cerebrospinal fluid; CT: computed tomography

Study/reference	Clinical setting	Intrathecal agent	Symptom onset	Key findings	Outcome
Doghmi et al., "Aseptic meningitis following a bupivacaine spinal anesthesia" [1]	31-year-old woman; cesarean delivery	Bupivacaine	10 hours after the procedure	Severe headache, neck stiffness, restlessness, and loss of consciousness; CT reported normal	Awake 24 hours after intubation; favorable clinical evolution
Oliveira et al., "Drug-induced aseptic meningitis following spinal anesthesia" [2]	Two cases following spinal anesthesia	Bupivacaine and ropivacaine	Shortly after the procedure	Meningitis symptoms with inflammatory CSF; viral and bacterial investigations negative	Rapid improvement without neurological sequelae
Tateno et al., "Bupivacaine-induced chemical meningitis" [3]	34-year-old woman; cesarean delivery	Intrathecal bupivacaine	Soon after cesarean delivery	CSF 3,040 cells/mm³, predominantly polymorphonuclear; protein 401 mg/dL; no organisms identified	Clinical condition and CSF normalized within 10 days
Present case	18-year-old primigravida; emergency cesarean delivery	Preservative-free hyperbaric bupivacaine 12.5 mg + fentanyl 25 µg	Somnolence at ≈4 hours; febrile meningeal syndrome at ≈6 hours	CSF ≈2,000 cells/µL with neutrophilic predominance; protein 3.05 g/L; preserved glucose; sterile culture	Complete neurological recovery; diplopia resolved by 1 month

Comparison with the available literature demonstrates several recurring characteristics: rapid onset after spinal anesthesia, prominent meningeal symptoms, neutrophilic CSF pleocytosis, negative bacterial cultures, and generally favorable neurological recovery [1-5]. Our case adds several noteworthy features, including very early neurological deterioration after obstetric spinal anesthesia, marked systemic neutrophilic leukocytosis, transient binocular diplopia with limitation of upward gaze, extensive exclusion of infectious and vascular causes, and documented near-complete normalization of CSF findings on repeat lumbar puncture.

This case also illustrates the potential value of serial CSF assessment when the initial diagnosis remains uncertain. The first CSF profile was highly inflammatory and could not safely distinguish bacterial from aseptic meningitis. By day 10, however, CSF leukocytes had decreased to 3 cells/µL (normal range: 0-5 cells/µL) and protein to 0.17 g/L (normal range: 0.15-0.45 g/L), with persistently sterile microbiological investigations. This rapid biological resolution, considered together with the clinical recovery, substantially strengthened the diagnosis of a self-limited aseptic inflammatory process.

This report has several strengths, including a clearly documented temporal relationship with spinal anesthesia, serial CSF examinations, extensive microbiological investigations, multimodal neuroimaging, and neurological follow-up demonstrating complete recovery. Nevertheless, important limitations should be acknowledged. No definitive biomarker exists for chemically induced meningitis, and the specific trigger could not be identified. Clinical improvement occurred in the setting of several empirical therapies, including antibacterial agents, corticosteroids, acyclovir, and temporary antituberculous treatment, making it impossible to attribute recovery solely to the natural history of the aseptic inflammatory process. In addition, detailed neuro-ophthalmological findings and verbatim radiological reports were not available. Therefore, the association with spinal anesthesia should be interpreted as strongly suggestive rather than proof of causality. Furthermore, the original CT and vascular imaging files were no longer available for independent review or inclusion in this report; therefore, the reported imaging findings were based on the available medical documentation.

From a practical perspective, acute fever and meningeal or encephalopathic symptoms following spinal anesthesia should initially be approached as a potential bacterial meningitis [6,7]. Appropriate microbiological sampling and empirical antimicrobial therapy remain essential [7]. Post-spinal aseptic meningitis should subsequently be considered when cultures and molecular studies remain negative, alternative neurological diagnoses have been excluded, and the clinical and CSF course demonstrates rapid resolution [1-5]. Recognition of this entity may ultimately help avoid unnecessarily prolonged anti-infective treatment once serious infectious causes have been adequately excluded.

## Conclusions

Post-spinal aseptic meningitis should be considered in patients who develop an acute febrile meningeal syndrome after spinal anesthesia, although bacterial meningitis must initially be excluded and treated empirically. In this case, negative microbiological investigations, rapid CSF normalization, and complete neurological recovery supported an aseptic inflammatory process. The transient binocular diplopia with limitation of upward gaze was an unusual neurological manifestation. Serial clinical and CSF assessment may help distinguish this rare complication from infectious meningitis.
